# Development of a protein-based system for transient epigenetic repression of immune checkpoint molecule and enhancement of antitumour activity of natural killer cells

**DOI:** 10.1038/s41416-019-0708-y

**Published:** 2020-01-21

**Authors:** Yoichi Teratake, Tomoki Takashina, Kenta Iijima, Tetsushi Sakuma, Takashi Yamamoto, Yukihito Ishizaka

**Affiliations:** 1 Department of Intractable Diseases, National Center for Global health and Medicine, 1-21-1 Toyama, Shinjuku-ku, Tokyo, 162-8655 Japan; 2 Division of Cancer Biology, Nagoya University Graduate School of Medicine, 65 Tsurumai-cho, Showa-ku, Nagoya, 466-8550 Japan; 3 Division of Integrated Sciences for Life, Graduate School of Integrated Sciences for Life, Hiroshima University, 1-3-1 Kagamiyama, Higashi-Hiroshima, Hiroshima, 739-8526 Japan

**Keywords:** Expression systems, Immune cell death, Cancer immunotherapy

## Abstract

**Background:**

Immune checkpoint blockade (ICB) therapy improved the prognosis of cancer patients, but general administration of ICBs occasionally induces side effects that include immune-related adverse events and tumour hyper-progression. Here, we established a protein-based system, by which endogenous expression of IC molecule in natural killer (NK) cells was transiently repressed on enhancement of their antitumour activity.

**Methods:**

A protein-based genome modulator (GM) system is composed of a transcription activator-like effector (TALE), DNA methyltransferase and a newly identified potent cell-penetrating peptide with nuclear-trafficking property named NTP. TALE was designed to target the promoter region of the programmed cell death-1 (*PD-1*) gene. After culturing human NK cells in the presence of NTP-GM protein, we examined endogenous *PD-1* expression and antitumour activity of the treated cells.

**Results:**

NTP-GM protein efficiently downregulated *PD-1* expression in NK cells with increased CpG DNA methylation in the promoter region. The antitumour activity of the treated NK cells was enhanced, and repeated intraperitoneal administrations of the treated NK cells attenuated tumour growth of programmed death-ligand 1-positive tumour cells in vivo.

**Conclusions:**

Because the incorporated NTP-GM protein was quickly degraded and negligible in the administered NK cells, the NTP-GM system could be an alternative option of an ICB without side effects.

## Background

The application of immune checkpoint blockade (ICB) therapy has had a great impact on the treatment of cancer.^1–3^ In US Food and Drug Administration-approved clinical trials, three different ICB antibodies targeting cytotoxic T-lymphocyte-associated antigen 4, programmed cell death protein 1 (PD-1) and programmed death-ligand 1 (PD-L1) produced favourable clinical outcomes.^4–7^ Clinical trials using these antibodies revealed that immune checkpoints have critical roles in tumour surveillance, and that antibody-based ICB therapies are a promising approach for cancer. However, there are several issues with the use of ICB therapies that must be addressed: various immune-related adverse events, including immune-mediated colitis, pneumonitis, and fatal type 1 diabetes mellitus, have been reported.^8,9^ In addition, there are a growing number of reports showing that tumour growth becomes hyperactive in some patients after treatment with nivolumab.^10–12^ Moreover, current antibody-based ICB therapies are not equally effective in patients, possibly because of the presence of different immune checkpoint molecules, such as lymphocyte-activation gene 3, T-cell immunoglobulin and mucin domain-containing 3 (TIM3), carcinoembryonic antigen-related cell adhesion molecule 1 (CEACAM1), cluster of differentiation 96 and CD112R.^13^ For complete tumour eradication, it would be necessary to combine multiple monoclonal antibodies, but such a combination might cause even more adverse events.

Under a model of PD-1 overexpression in exhausted T cells in cancer conditions, it has been suggested that the promoter region of *PD-1* is demethylated,^14^ providing a possible novel approach for ICB therapy, by which DNA methylation of the promoter is upregulated. Recently, Siddique et al.^15^ reported that a chimeric molecule composed of DNA methyltransferase subtypes 3A and 3L (DNMT3A/3L), when fused to a zinc-finger motif, a DNA-binding module that was developed in the first generation of genome-editing systems, could be utilised for site-specific DNA methylation of a target site.^15–17^ DNMT3A catalyses DNA methylation, whereas DNMT3L has no catalytic activity, but greatly enhances the catalytic activity of DNMT3A.^18^

In our previous work, we identified a cell-penetrating peptide (RIFIHFRIGC, amino acids depicted in single letters) with nuclear- trafficking properties (NTP: nuclear-trafficking peptide),^19^ and established a protein-based artificial transcription factor (ATF) system by combining NTP with a chimeric protein of VP64 and a transcription activator-like effector (TALE), which functions as a DNA-binding module.^20,21^ As TALEs are readily designable and have low toxicity, we generated a functional NTP–ATF protein that could upregulate the expression of the *microRNA302/367* cluster gene, and successfully established mouse-induced pluripotent stem cell-like clones by using an NTP–ATF protein. Moreover, we generated chimeric mice with the established clones, indicating that the NTP–ATF system is safe without negative effects on organogenesis.^19^

In this study, we applied NTP to a protein-based genome modulator (GM) system, in which VP64 was replaced with DNMT3A/3L that functions as a repressor of the gene of interest (NTP-GM). Currently, we selected the *PD-1* gene as a target of the NTP-GM system and proved that treatment with NTP-GM protein transiently increased DNA methylation of the *PD-1* promoter region and reduced the expression of endogenous *PD-1* mRNA. In peripheral blood mononuclear cells (PBMCs) and natural killer (NK) cells, *PD-1* mRNA expression was reduced to ~50% of the control level. Concomitantly, the cytotoxic activity of NK cells was transiently upregulated in vitro and in vivo. The function of NTP-GM protein depended exclusively on methyltransferase activity, because NTP-GM protein that possessed a mutant DNMT3A with no DNA methylation activity had no effects on *PD-1* expression. Taken together with the observation that NTPGM protein, once incorporated into human cells, is degraded quickly and free of apparent toxic effects, the data implicate that our system could provide an effective and safe option for ICB therapy.

## Methods

### Cell culture

MOLT-4, Jurkat, CEM and RPMI8226 cells were obtained from the RIKEN Cell Bank and maintained in RPMI-1640 (Gibco, Gaithersburg, MD) medium with 10% foetal bovine serum (FBS) (Gibco, Gaithersburg, MD) at 37 °C in 5% CO_2_. SKOV-3/Luc cells (Cell Biolabs, San Diego, CA), a human ovarian cancer cell line with an exogenous luciferase gene, were maintained in Dulbecco’s modified Eagle’s medium (Gibco, Gaithersburg, MD) with 10% FBS at 37 °C in 5% CO_2_. The experimental protocol using healthy donors was approved by the Internal Review Board of the National Centre for Global Health and Medicine (Ref. No. NCGM-A-000268-00). The peripheral blood was isolated from healthy donors who gave written informed consent, and PBMCs were prepared, as described previously.^19^ For preparation of NK cells, PBMCs were expanded for 7 days using an NK Cell Activation/Expansion Kit (Miltenyi Biotec, Bergisch Gladbach, Germany), and NK cells were isolated with an EasySep Human NK Cell Isolation Kit (Veritas, Tokyo, Japan). Then, NK cells were cultured for an additional 7 days in the presence of NTP-GM protein.

### Construction of plasmid DNA

pEU-NTP-GM vector is derived from pEU-01 vector (CellFree Sciences, Kanagawa, Japan) with additional DNA fragment encoding glutathione *S*-transferase (GST) as a tag and NTP.^19^ A 1.2-kb synthesised DNA fragment encoding the 3′ region of TALE and the catalytic domain of DNA methyltransferase 3α (DNMT3A) complementary DNA (cDNA)^17^ (Eurofins Genomics, Tokyo, Japan) was inserted into the pEU-E01-GST-NTP vector (pEU-E01-GST-NTP-DNMT3A). A DNA fragment encoding DNMT3L, encompassing amino acids 121–386^17^ with *Bst*BI and *Nhe*I sites at the 5′ and 3′ termini, respectively, was synthesised (Eurofins Genomics, Tokyo, Japan) and inserted into pEU-E01-GST-NTP-DNMT3A (pEU-E01-GST-NTP-DNMT3A/3L). TALE molecules designed to target the *PD-1* promoter sequence (C17 bp: TCCCCCAGCACTGCCTC; D17 bp: TCCCTTCAACCTGACCT; L17 bp: TCCAGGCATGCAGATCC; M17 bp: TCCAGACCCCTGGCTCT; N17 bp: TCCCTCCAGACCCCTGG) were assembled as described previously^22^ and inserted into the *Xho*I site of pEU-E01-GST-NTP-DNMT3A/3L, which was present at the junction of NTP and DNMT3A/3L. The obtained vector was named pEU-NTP-GM. This vector had a PreScission protease cleavage site between GST and NTP. The sequences of D17 and SCR TALE cDNAs are shown in Supplementary Table 1.

A bacterial expression vector was originally derived from the pCold-I vector (Takara Bio, Shiga, Japan), by which the expression of recombinant proteins is tightly suppressed at 37 °C, whereas it is induced by changing the temperature to 15 °C. First, to remove the amino-terminal (His)_6_ tag, which was present in the 5′ region of the pCold-I vector, an *Nco*I site was generated at the transcription start site by quick-change mutagenesis using PCR with mutated primers, followed by digestion with *Dpn*I (Takara Bio, Shiga, Japan) and bacterial transformation. Then, a DNA fragment (5′-CATGAGGATCTTCATCCACTTCCGGATCGGCTGTGAAAACCTGTATTTCCAATCTCTCGAGGATATCGTTTAAAC-3′) encoding NTP (the underlined sequence encodes RIFIHFRIG) and a part of the 5′ region of TALE was inserted into the *Nco*I and *Eco*RV sites (pCold-I-NTP).^19^ For purification of NTP-GM proteins, an oligonucleotide (5′-TAGAGGTCTTGTTCCAGGGACCACACCACCACCATCATCAC-3′) encoding the PreScission Protease recognition site and (His)_6_ tag was ligated to the 3′ region of pCold-I-NTP. Then, pCold-I-NTP-DNMT3A/3L was generated by introducing a DNMT3A/3L cDNA fragment to pCold-I-NTP. Finally, a *Xho*I TALE fragment was inserted into the *Xho*I site, which was located between NTP and DNMT3A/3L in the pCold-I-NTP-DNMT3A/3L, and the obtained plasmid was named pCold-I-NTP-DNMT3A/3L-(His)_6_ (pCold-I-NTP-GM). pCold-I-NTP-SCR was similarly generated using a TALE that would recognise TGGCTCCCACACCCTCG, a randomly designed sequence. pCold-I-NTP-GM-DNMT3A^E752A^/3L was generated from the pCold-I-NTP-GM plasmid using the QuickChange Site-Directed Mutagenesis Kit (Agilent Technologies, California, USA), following the manufacturer’s protocol. To introduce the mutation, we used the following pair of oligonucleotide primers: forward primer, 5′-CTTCTTCTGGCTCTTTGCGAATGTGGTGGCCATG-3′; reverse primer, 5′-CATGGCCACCACATTCGCAAAGAGCCAGAAGAAG-3′.

### Expression and purification of NTP-GM proteins

As initial screening for the TALE clones, pEU-NTP-GM proteins were prepared using a WEPRO 7240G Expression Kit (CellFree Sciences), as described previously.^19^ Each protein was treated with PreScission protease (GE Healthcare, Illinois, USA) (Supplementary Fig. S1A), and the cleaved product was subjected to a DNA methylation assay. For bacterial expression, the pCold-I vector was used, by which the expression of NTP-GM recombinant proteins could be tightly regulated by cold shock at 15 °C in BL21 (DE3) cells (Takara Bio, Shiga, Japan). NTP-GM proteins were purified by three column chromatography steps that included an affinity column with an anti-TALE monoclonal antibody.^19^ After overnight induction, cells were collected by centrifugation for 15 min at 6000 r.p.m. and suspended in a lysis buffer composed of 20 mM sodium phosphate, pH 7.6, 10% glycerol, 500 mM NaCl, 10 mM mercaptoethanol, 20 mM imidazole, 0.1% Nonidet P-40 (NP-40), 1 mM phenylmethylsulfonyl fluoride (PMSF), and 1.5 mg/mL lysozyme (Wako Pure Chemical Industries, Osaka, Japan). After incubation for 30 min on ice, cells were sonicated and centrifuged for 30 min at 18,000 r.p.m. The supernatant was loaded onto a Ni-NTA column (Thermo Fisher Scientific, Waltham, MA), and the proteins were eluted with 250 mM imidazole. As the second purification step, the recovered proteins were loaded onto a Heparin HP column (GE Healthcare, Illinois, USA) and eluted with a linear gradient of 50 mM to 1 M NaCl in a buffer with 10% glycerol, 50 mM Tris-HCl, pH 8.0 and 0.1 mM dithiothreitol (DTT; Sigma-Aldrich, St. Louis, MO). Finally, recovered fractions were incubated for 2 h with CNBr Sepharose (GE Healthcare, Illinois, USA) that was conjugated with an anti-TALE antibody at 4 °C.^19^ After extensive washing with phosphate-buffered saline (−) (PBS(−)), which was prepared by endotoxin-free water just before use, NTP-GMs were eluted using 0.1 M 4-(2-hydroxyethyl)-1-piperazineethanesulfonic acid (HEPES, pH 2.5; Sigma-Aldrich, St. Louis, MO). Immediately after elution, the eluate was neutralised by 1 M HEPES buffer (pH 8.0). The concentration of endotoxin in each sample was <0.03 EU/mL, as examined using an Endospecy ES-50M Kit (Seikagaku, Tokyo, Japan). Toxic effects of NTP-GM-D17 on MOLT-4 cells or PBMCs were assessed by trypan blue staining assay (MP Biomedicals, Tokyo, Japan) or cytotoxicity LDH Assay Kit-WST (Dojindo Laboratories, Tokyo, Japan), in which lactate dehydrogenase (LDH) in the culture supernatant was measured. Assays were carried out according to the manufacturer’s protocol.

### DNA methylation analysis

A 2.2-kb DNA fragment encompassing the human *PD-1* promoter was cloned into pBluescriptII and incubated with NTP-GM or CpG methyltransferase (M.SssI) (New England BioLabs, Ipswich, MA) in a reaction buffer of 50 mM NaCl, 10 mM Tris-HCl (pH 8.0), 10 mM MgCl_2_ and 1 mM DTT. After incubation for 3 h at 37 °C, the DNA was subjected to a Cells-to-CpG Bisulfite Conversion Kit (Applied Biosystems, Foster City, CA), and the targeted loci (CR-C and CR-B) were amplified by PCR using KOD-Multi&Epi-® (Toyobo, Osaka, Japan), followed by sequencing. Primers were designed to amplify an approximate 250–300-bp fragment of the CpG islands in CR-C and CR-B. Nucleotide sequences of primers for CR-C were 5′-GGGAGTGGTTTTTTGTTTATAAA-3′ (forward primer) and 5′-AAAATCCAATACCTAAACCTAACTAAC-3′ (reverse primer), whereas those for CR-B were 5′-TTAATTTGATTTGGGATAGTTTTTTTT-3′ (forward primer) and 5′-CCCTCCAAACCCCTAACTCTAAAAC-3′ (reverse primer). Methylation frequency was measured by using Quantification Tool for Methylation Analysis.^23^

MOLT-4 cells, PBMCs and NK cells were seeded onto a 96-well plate at a density of 2.0 × 10^5^ cells/mL. After 24 h, 1–20 nM NTP-GMs were added to each well once a day for 5 days. On the day following the last addition of the proteins (day 6 after the initial addition), the cells were subjected to analysis.

### Quantitative reverse-transcription PCR analysis of gene expression

Endogenous expression was analysed as described previously.^19^ *PD-1* mRNA levels were normalised according to *GAPDH* mRNA levels. The primers used for quantitative reverse-transcription PCR are listed in Supplementary Table 2.

### Flow cytometry analysis

Fluorescence-activated cell sorting (FACS) analysis was performed as described previously.^19^ Fixed cells were reacted with an anti-CD3-FITC antibody (1:100; BD Biosciences, Franklin Lakes, NJ), anti-PD-1-PE antibody (1:100; BD Biosciences, Franklin Lakes, NJ), anti-CD56-APC antibody (1:100; BioLegend, San Diego, CA) and anti-interferon-γ (IFN-γ)-PE antibody (1:100; BD Biosciences, Franklin Lakes, NJ). Before anti-IFN-γ intracellular staining, the cells were treated with 0.5% Tween-20 in PBS (−).

### Measurement of cytotoxic activity

PBMCs were treated with NTP-GM for 5 days and used as effector cells for the cytotoxicity assay. RPMI8226 cells, a human multiple myeloma cell line, were used as target cells. To identify the target cells, they were pre-labelled with 5 μM of 5- or 6-(*N*-succinimidyloxycarbonyl)fluorescein 3′, 6′-diacetate (Dojindo Molecular Technologies, Tokyo, Japan) for 30 min at 37 °C in the dark. After co-culture of RPMI8226 cells (1.0 × 10^5^ cells) and a threefold excess number of PBMCs (3.0 × 10^5^ cells) for 16 h, the cells were rinsed with PBS(−), reacted with PerCP-Cy™5.5 Annexin V (1:20; BD Biosciences, Franklin Lakes, NJ) in an Annexin V-binding buffer (10 mM HEPES [pH 7.4], 140 mM NaCl and 2.5 mM CaCl_2_) and analysed using a FACSVerse Flow Cytometer (BD Biosciences, Franklin Lakes, NJ). NK cells (3.0 × 10^5^ cells), which were treated with NTP-GM-D17 for 5 days, were co-cultured for 16 h with 1.0 × 10^5^ SKOV-3/Luc cells in 0.2-mL medium. LDH in the culture supernatant was measured by using Cytotoxicity LDH Assay Kit-WST.

### Construction of the xenograft model and evaluation of antitumour effects

All protocols of animal experiments were approved by the committee of Institutional Animal Care and Use (Ref. No. 19055). All mice were housed in an air-conditioned animal room at 23 ± 2 °C. Its relative humidity was 40–60% under specific-pathogen-free conditions with 12-h light/dark cycle (light on at 8:00 a.m.). A standard laboratory diet, CE-2 (CLEA Japan, Tokyo, Japan), and filtered tap water containing 10-p.p.m. chlorine were supplied ad libitum. All mice were kept in standard plastic cages [Clean S-PSF (w207 × d314 × h128), CLEA Japan], which contained wood shavings for bedding materials. Eight-week-old female nonobese diabetic/severe combined immunodeficiency (NOD-SCID) mice (CLEA) of 25 ± 2 g in body weight were purchased, and a maximum of two mice were maintained in one cage.

In the current study, three sets of experiments in vivo were conducted using a total of 38 mice in the laboratory of animal experiments (Exp. 1–3). Exp. 1 was carried out for evaluating the antitumour effects of PBMCs that had been treated with NTP-GM-D17, whereas other two experiments (Exp. 2 and 3) were for evaluating the antitumour effects of NK cells. In each experiment, 10, 12 and 16 mice were first enrolled, anaesthetised and subcutaneously inoculated with 1.0 × 10^6^ SKOV-3/Luc cells on the back. Anaesthesia was carried out with isoflurane (Zoetis, Tokyo, Japan). After 7 days of the inoculation, tumour formation was confirmed by measuring chemiluminescent signals that were generated by *luciferase* gene inserted in SKOV-3/Luc cells and d-luciferin as a substrate (Promega, Madison, WI). Under anaesthesia, mice were intraperitoneally injected with 3 μg of d-luciferin and subjected to analysis by the IVIS imaging system (Caliper Life Sciences, Waltham, MA). Mice, the chemiluminescent signals of which were negative or weak, were excluded and continuously maintained in SPF. In Exp. 1, 16 mice were first enrolled and 12 mice with definite chemiluminescent signals were randomly divided into two groups. On day 8 after the inoculation (on the next day of the IVIS imaging analysis), six of them were intraperitoneally injected with 5.0 × 10^5^ PBMCs, which were treated with NTP-GM-SCR (control cells). As a test group, six mice were intraperitoneally injected with 5.0 × 10^5^ PBMCs, which were treated with NTP-GM-D17 (treated cells). Injections of PBMCs were repeated on days 11, 14, 17, 20, 23 and 26, whereas imaging analysis under anaesthesia was performed on days 14, 21 and 28. After imaging analysis, mice were sacrificed under anaesthesia by cervical dislocation.

In Exp. 2, ten mice were first enrolled and six mice with definite chemiluminescent signals were subjected to similar procedures described in Exp. 1. As control, three mice were injected with 5.0 × 10^5^ control NK cells, whereas three mice were injected with the same number of the treated NK cells as a test group. To reduce the number of mice used for in vivo experiments, we did not include additional control experiments, in which non-treated PBMCs or non-treated NK cells were used.

In Exp. 3, 12 mice were first enrolled and 8 mice with definite chemiluminescent signals were used for the experiments. As control, four mice were injected with 5.0 × 10^5^ control NK cells, whereas another four mice were injected with the treated NK cells. Repeated injections and IVIS imaging analysis were carried out similarly as described in Exp. 1. In Exp. 3, mice were sacrificed on day 28 under anaesthesia by cervical dislocation, and tumour tissues were excised for further analysis. Data of chemiluminescent signals obtained by Exp. 2 and 3 (*n* = 7) were simultaneously subjected to statistical analysis.

To evaluate the adverse effects of treated NK cells, we injected the treated NK cells into two mice, which gave no chemiluminescent signals in Exp. 2, and started monitoring whether injected NK cells form tumours. In 15 weeks after the last injection of the cells, mice were analysed. In this experiment, one non-treated mouse was included.

### Tissue preparation

Tissues were soaked in 30% (wt/vol) sucrose followed by freezing in Tissue-Tec OCT compound (Sakura Finetek, Tokyo, Japan). For histological examination, tissue sections were cut from the frozen block of 5-μm-thick sections, dried and subjected to analysis.

### Immunohistochemical analysis and detection of apoptotic cells

Immunohistochemical analysis was performed as described previously.^19^ Antibodies to Ki67 (Sigma-Aldrich, St. Louis, MO), CD56 (Abcam, Cambridge, UK) and cleaved caspase-3 (Cell Signalling Technology, Danvers, MA) were used as primary antibodies. Alexa 488- or 555-conjugated secondary antibodies (Thermo Fisher Scientific, Waltham, MA) were used for detection. After staining nuclei with Hoechst 33258 (Thermo Fisher Scientific, Waltham, MA), the specimens were analysed with a BZ-X700 microscope (Keyence Corporation, Osaka, Japan) using Bzx Analyser (Keyence Corporation, Osaka, Japan). Apoptotic cells were detected by terminal deoxynucleotidyl transferase dUTP nick-end labelling (TUNEL) using an In Situ Cell Death Detection Kit (Roche, Basel, Switzerland), according to the manufacturer’s protocol.

### Western blot analysis

PBMCs were suspended in sampling buffer (2% sodium dodecyl sulfate (SDS), 300 mM Tris-HCl (pH 6.8) and 10% glycerol) and boiled for 10 min at 100 °C. After SDS-PAGE (SDS-polyacrylamide gel electrophoresis), proteins were transferred to a polyvinylidene difluoride membrane (Millipore, Burlington, MA). The membrane was incubated for 1 h at room temperature in blocking solution (5% skim milk, 0.1% Tween-20 in Tris-buffered saline). After blocking, the membrane was incubated with anti-TALE (1:1000),^19^ anti-α-tubulin (1:2000, Proteintech, Illinois, USA), anti-histone H3 (1:1000, FUJIFILM, Osaka, Japan) and β-actin (1:1000, Sigma-Aldrich, St. Louis, MO) for 2 h at room temperature. After washing, the membrane was incubated for 1 h in PBS(−) containing goat anti-mouse immunoglobulin G (IgG) conjugated with horseradish peroxidase (1:1000, GE Healthcare, Illinois, USA). Immunoblots were detected by using ECL Prime Western Blotting Detection Reagent (GE Healthcare, Illinois, USA) and imaging system LAS4000 (GE Healthcare, Illinois, USA).

To detect NTP-GM protein in the nucleus, nuclei of PBMCs were prepared according to the described procedures.^22^ Briefly, PBMCs were treated with 20 nM of NTP-GM-SCR for 12 h, washed with PBS(−) and resuspended in 500 μL of solution A, which was composed of 10 mM HEPES (pH 7.9), 10 mM KCl, 0.1 mM EDTA, 1 mM DTT, 0.5 mM PMSF and 1% NP-40. Cells were incubated on ice for 15 min, followed by centrifugation for 5 min at 15,000 r.p.m. The supernatant and pellet were recovered as cytoplasmic and nuclear fractions, respectively. Isolated nuclei were resuspended in 150 μL of solution B of 20 mM HEPES (pH 7.9), 0.42 mM NaCl, 1 mM EDTA, 1 mM DTT and 1 mM PMSF.

### Gene knockout of PD-L1

Gene disruption was carried out by CRISPR–Cas9 (clustered regularly interspaced short palindromic repeats—CRISPR-associated proteins) system. PD-L1 single-guide RNA nucleotides were cloned into *Bgl*II and *Hind*III sites of pSUPER vector (Oligoengine, Seattle, WA) (oligonucleotide sequences are shown in Supplementary Table 3). Constructed vector and pMJ920 (Addgene, Cambridge, USA) plasmid, containing the Cas9 endonuclease coding region, were transfected to SKOV-3/Luc cells by FuGENE6 Transfection Reagent (Promega, Madison, WI), according to the manufacturer’s protocol. Knockout clones were isolated by a single-cell dilution cloning and identified by flow cytometry analysis for cell surface PD-L1.

### Statistical analysis

Quantitative real-time PCR and FACS analysis data were analysed using Student’s *t* test. Data from the DNA methylation assay were analysed by the Mann–Whitney *U* test. A difference with a *P* value <0.05 was defined as statistically significant. Data are depicted by mean values ± standard deviation (SD).

## Results

### Identification of NTP-GM protein that induces DNA methylation in the *PD-1* promoter region

**Fig. 1:**
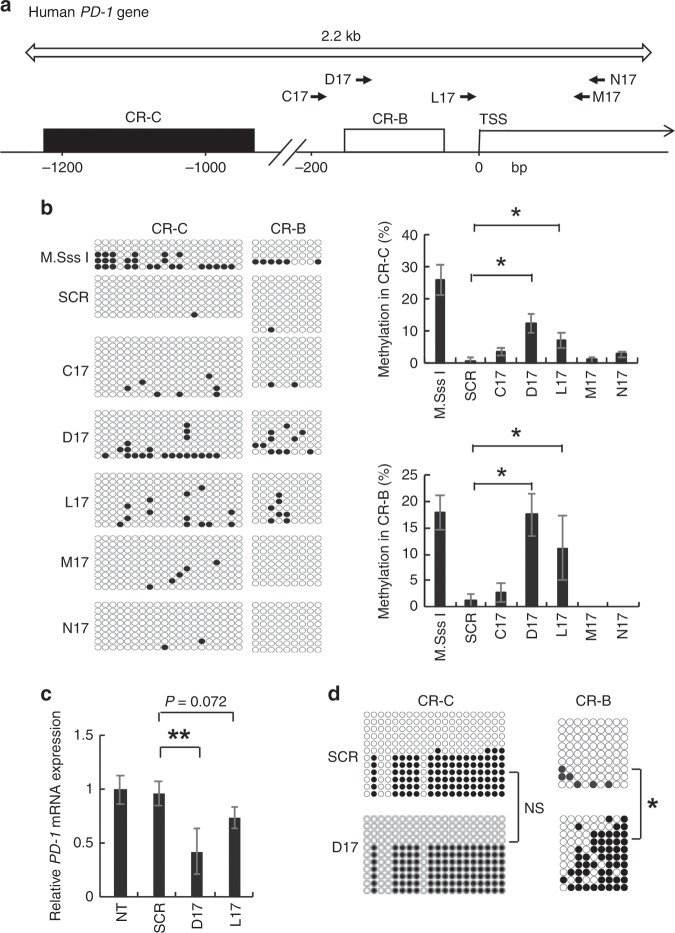
Identification of NTP-GM proteins targeting PD-1. **a** Design of TALEs in the promoter region of the human *PD-1* gene. Arrows indicate sites targeted by NTP-GM proteins (C17, D17, L17, M17 and N17). C17, D17 and L17 TALEs were designed in the sense orientation, whereas M17 and N17 were designed in the antisense orientation. Open and filled squares indicate CR-C and CR-B, respectively. TSS, transcription start site. Open arrow indicates the region amplified for DNA methylation analysis. **b** Bisulfite sequence analysis of the *PD-1* promoter region. NTP-GM proteins were expressed and purified using a wheat germ cell-free protein expression system (see Methods and Supplementary Figure S1), and DNA methylation activity was assayed in vitro. Each horizontal alignment represents the sequence data of individual clones (left panel). Closed and open circles indicate methylated and unmethylated CpGs, respectively. One representative result of three independent experiments was shown in the left panel. Summarised data depicting integrated data of 12–20 independent clones are shown in the right panel. M.SssI was used as a positive control. Values represent the mean ± SD. *P < 0.01. SCR, scramble protein as control. **c** NTP-GM-D17 efficiently repressed *PD-1* mRNA expression. MOLT-4 cells were treated for 5 days with 1 nM NTP-GM protein. Expression levels were normalised to *GAPDH* mRNA. Data analysed on three independent experiments were normalised by non-treated samples (NT) and depicted as the mean ± SD. **d** Bisulfite sequence analysis of CR-C and CR-B in MOLT-4 cells that were treated for 5 days with 1 nM NTP-GM. One representative result of three independent experiments was shown. *P < 0.01; **P < 0.05; NS, not significant.

As the *PD-1* promoter contains two CpG-rich regions, clusters of region C (CR-C) and B (CR-B),^14^ we designed five TALEs targeting 17-bp sequences present in the −168 to +128 bp region of the *PD-1* gene (C17, D17, L17, M17 and N17 shown in Fig. 1a: target sequences are described in the ‘Methods' section).^24^ Each NTP-GM protein, which is composed of NTP, TALE and DNMT3A/3L, was expressed in a cell-free system using wheat germ extract (see Supplementary Fig. S1A and Methods), purified and subjected to an in vitro DNA methylation assay. Repeated DNA methylation assays done using a PCR-amplified DNA fragment encompassing the target sequence (open arrow in Fig. 1a) revealed that NTP-GM protein with TALE-D17 (NTP-GM-D17) and TALE-L17 had definite CpG DNA methylation activity (Fig. 1b, the left panel depicts one representative result and the right panel shows the integrated data from three independent experiments. *P < 0.01). In contrast, control NTP-GM protein, which had a scrambled TALE (17 bp: TGGCTCCCACACCCTCG) (NTP-SCR), did not.

We then studied the effects of NTP-GM-D17 and NTP-GM-L17 on a human leukaemia MOLT-4 cell line, which expresses a definite level of *PD-1* mRNA with a relatively low level of methylated DNA in the *PD-1* promoter region, compared with the other two cell lines examined (Jurkat and CEM cells) (Supplementary Fig. 1B, C). By treating the cells with 1 nM of each NTP-GM protein once a day for 5 days, NTP-GM-D17 and NTP-GM-L17 decreased the expression of *PD-1* mRNA to ~50% and 80% of the control level, respectively (Fig. 1c, **P < 0.05). On the basis of repeated experiments showing that NTP-GM-D17 repressed *PD-1* expression and induced DNA methylation in CR-B (Fig. 1d, *P < 0.01), we selected NTP-GM-D17 for further experiments.

### Preparation of NTP-GM protein using a bacterial expression system

**Fig. 2:**
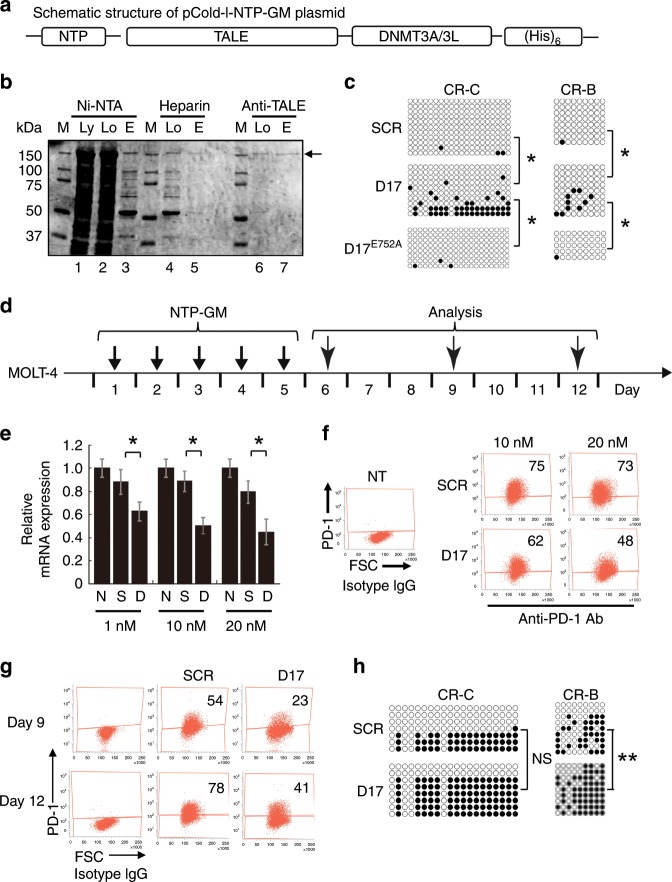
Large-scale preparation of NTP-GM-D17 and effects of NTP-GM-D17 on *PD-1* expression and DNA methylation of the *PD-1* promoter region. **a** Schematic structure of pCold-I-NTP-GM. The vector contained NTP, TALE, DNMT3A/3L and a (His)_6_ tag for purification. **b** CBB staining patterns of purification of NTP-GM-D17 protein. The arrow indicates purified NTP-GM-D17. M, marker; Ly, initial lysate; Lo, loaded sample; E, elution. **c** Bisulfite sequence analysis of CR-C and CR-B. Purified NTP-GM-D17 was subjected to an in vitro DNA methylation assay. *P < 0.01. **d** Experimental protocol for the treatment of MOLT-4 cells with NTP-GM-D17. After 5 days of treatment with NTP-GM-D17, MOLT-4 cells were cultured for another 7 days without NTP-GM-D17. On days 6, 9 and 12 after the initial addition of the protein, the cells were harvested and subjected to analysis. **e** NTP-GM-D17 reduces the expression of *PD-1* mRNA. MOLT-4 cells were treated for 5 days with 1, 10 and 20 nM NTP-GM-D17. On day 6 after the initial addition of the protein, the cells were harvested and subjected to quantitative real-time PCR analysis. *PD-1* mRNA expression levels were normalised to the level of *GAPDH* mRNA. Data are shown as the mean ± SD (*n* = 3). *P < 0.01. **f** FACS analysis of PD-1^+^ MOLT-4 cells. Treatment with 10 or 20 nM NTP-GM-D17 reduced the number of PD-1^+^ cells. Each number indicates the percentage of PD-1^+^ cells. **g** Dot plot of one representative result of FACS analysis of PD-1^+^ MOLT-4 cells. Cells were treated for 5 days with 20 nM NTP-GM-D17 and harvested on days 9 and 12. **h** NTP-GM-D17 induced DNA methylation of the *PD-1* promoter region that persisted for 7 days. Data from bisulfite sequence analysis of CR-C and CR-B are shown. MOLT-4 cells treated for 5 days with NTP-GM-D17 and harvested on day 12. **P < 0.05; NS, not significant.

To perform larger-scale experiments, we changed to a bacterial protein-expressing system by using the pCold-I vector and BL21 cells, in which the expression of a recombinant protein is induced by cold shock at 15 °C. Recombinant proteins were tagged with (His)_6_ at the C terminus (Fig. 2a), and purified by three steps using a Ni-NTA affinity column, heparin column and affinity column with a monoclonal anti-TALE antibody^19^ (Fig. 2b). The purity of the recovered protein was >90%, as judged by Coomassie brilliant blue (CBB) staining (Fig. 2b, lane 7, arrow). In an in vitro DNA methylation assay, NTP-GM-D17 upregulated CpG methylation in CR-C and CR-B (Fig. 2c, *P < 0.01). In striking contrast, DNA methylation was not increased by a variant of NTP-GM-D17 that possessed DNMT3A^E752A^, a mutant lacking catalytic activity^17^ (Fig. 2c).

A dose–response experiment revealed that treatment with 20 nM NTP-GM-D17 for 5 days (an experimental protocol depicted in Fig. 2d) exerted the most potent suppressive effects on *PD-1* mRNA expression (Fig. 2e, *P < 0.01) and the number of PD-1-positive (PD-1^+^) cells (Fig. 2f), and we decided to use 20 nM NTP-GM-D17 and NTP-SCR as control, for further experiments.

To apply this system to cellular adoptive immunotherapy, it is important to know how long the repressive effects of NTP-GM-D17 persist for after the last treatment. To examine this, cell culture was continued after 5-day treatment with NTP-GM-D17, and the cultured cells were sampled serially on days 9 and 12 and subjected to analysis. Notably, the number of *PD*-1^+^ cells was decreased in both samples (Fig. 2g) with an increase of DNA methylation in CR-B in the cell sample prepared on day 12 (Fig. 2h, **P < 0.05).

### NTP-GM-D17 decreases PD-1^+^ populations in PBMCs

**Fig. 3:**
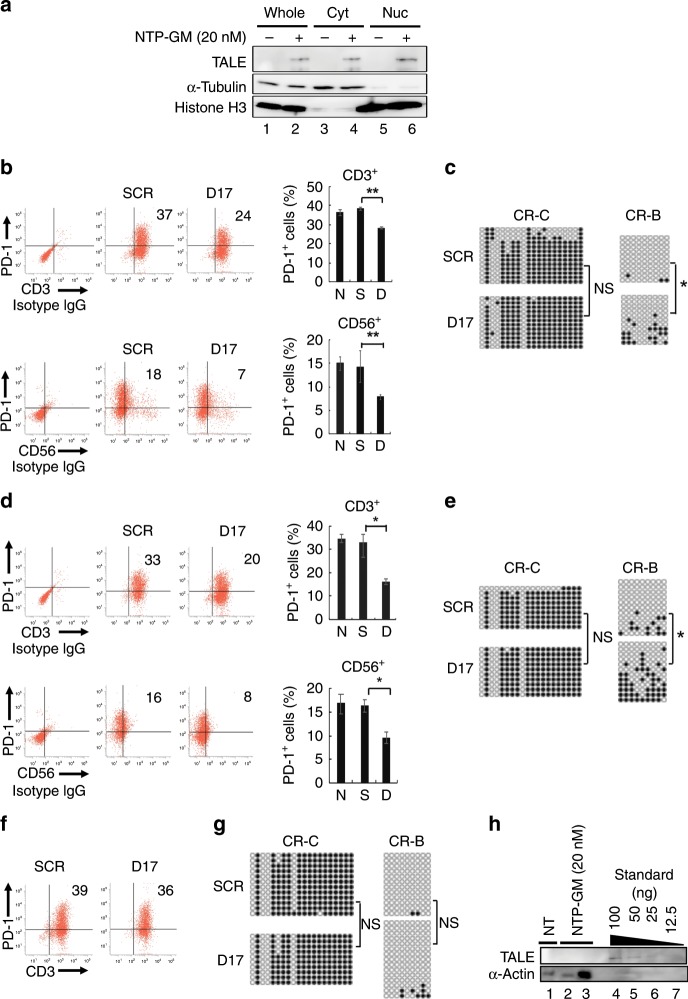
Effects of NTP-GM-D17 on PBMCs. **a** NTP-GM protein is translocated to the nucleus. PBMCs (1.0 × 10^7^ cells) were treated with 20 nM NTP-GM-D17 for 12 h and cellular fractionation was performed. Anti-α-tubulin and anti-histone H3 antibodies were used for detecting proteins localised in cytosolic or nuclear fractions, respectively. Whole, whole lysate. Cyt, cytoplasmic fraction. Nuc, nuclear fraction. **b** NTP-GM-D17 reduces the number of PD-1^+^/CD3^+^ and PD-1^+^/CD56^+^ cells. Dot plot from one representative FACS analysis (left) and a graph showing integrated data from three independent experiments (right) are shown. Values represent the mean ± SD (*n* = 3). **c** Bisulfite sequence analysis of the *PD-1* promoter region. Results for CR-C and CR-B in PBMCs, which were harvested on day 6 (see Fig. 2d) are shown. DNA methylation of CR-B was significantly increased by NTP-GM-D17. *P < 0.01; NS, not significant. **d** NTP-GM**-**D17 reduces the number of PD-1^+^/CD3^+^ and PD-1^+^/CD56^+^ cells on day 9 (see an experimental protocol shown in Fig. 2d). A graph of integrated data from three independent experiments was shown in the right panel. Values represent the mean ± SD (*n* = 3). **e** DNA methylation persists for 4 days after the last addition of NTP-GM-D17. Bisulfite sequence analysis of CR-C and CR-B was performed using PBMCs that were harvested on day 9. *P < 0.01; NS, not significant. **f** Effects of NTP-GM-D17 were cancelled on day 12. A representative result was shown. **g** Increased DNA methylation was restored on day 12. Data of bisulfite sequence analysis done on day 12 are depicted. NS, not significant. **h** Incorporated NTP–GP protein is degraded quickly. After treatment of PBMCs with 20 nM NTP-GM protein for 5 days, the cells were harvested on the following day, and whole-cell extracts of 1.0 or 5.0 × 10^5^ cells (lanes 2 and 3) were subjected to western blot analysis. As control, 100 (lane 4), 50 (lane 5), 25 (lane 6) and 12.5 ng (lane 7) of NTP-GM protein was loaded onto the same SDS-PAGE gel.

After confirming the effects of NTP-GM-D17 on primary human PBMCs (Supplementary Fig. S2A, B), we expanded PBMCs using interleukin-2, an immobilised anti-CD3 antibody, and anti-CD16 antibody. Consistent with the previous reports,^25,26^ the population of PD-1^+^ cells increased from 3 to 47% during expansion for 14 days (Supplementary Fig. S2C). After confirming that NTP-GM protein, when added to the culture medium of the expanded PBMCs, was translocated to the nucleus (Fig. 3a, lane 6), we examined the effects of NTP-GM-D17 on the expanded NK cells by the same experimental protocol as for MOLT-4 cells (Fig. 2d). The number of cells double positive for PD-1 and CD3 (PD-1^+^/CD3^+^ cells) decreased from 37 to 24%, whereas PD-1^+^/CD56^+^ cells decreased from 18 to 7% (Fig. 3b, **P < 0.05). Consistently, CpG DNA methylation was decreased in the CR-B (Fig. 3c, *P < 0.01). Again, mutant NTP-GM-D17 with DNMT3A^E752A^, which lacks DNA methylation activity, did not repress *PD-1* expression (Supplementary Fig. S2D). Fortunately, we observed no apparent cytotoxic effects of 1–20 nM NTP-GM-D17 on expanded PBMCs (Supplementary Fig. S2E).

Importantly, the suppression of *PD-1* expression was sustained on both CD3^+^ and CD56^+^ cells for an additional 4 days after the last addition of NTP-GM-D17 (Fig. 3d, day 9 in Fig. 2d, Supplementary Fig. S2F). Increased CpG DNA methylation was also detected in the treated PBMCs (Fig. 3e, *P < 0.01). In contrast, however, *PD-1* expression was restored in the cells collected 7 days after the last treatment with NTP-GM-D17 (Fig. 3f, day 12 in Fig. 2d), with concomitant recovery of the DNA demethylation to the non-treated level (Fig. 3g). Western blot analysis done on the next day of the last treatment of 20 nM NTP-GM protein detected no definite signals of the incorporated protein in the cells (Fig. 3h, lanes 2 and 3). These data implied that the downregulation of *PD-1* expression is maintained for several days, once its promoter region is methylated by NTP-GM protein.

### Downregulation of PD-1 enhances the antitumour activity

**Fig. 4:**
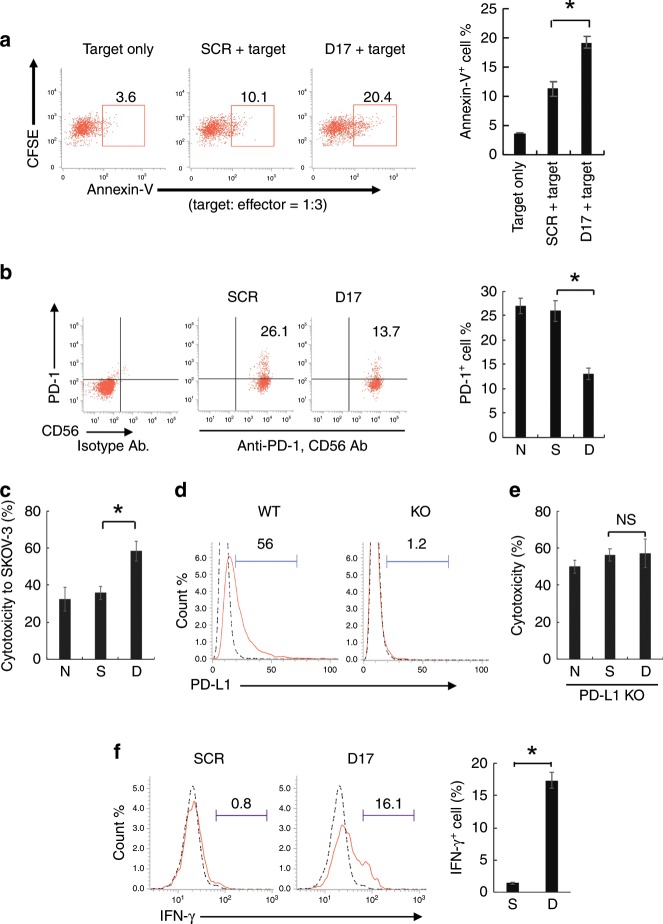
Treatment of NTP-GM-D17 enhanced antitumour activity. **a** Enhancement of antitumour activity by PBMCs. PBMCs were treated with NTP-GM-D17 and mixed with 5- or 6-(*N*-succinimidyloxycarbonyl)fluorescein 3′,6′-diacetate (CFSE)-labelled RPMI8226 cells, and the number of annexin V^+^ cells were analysed. The experiments were performed with target cells and treated with PBMCs at a ratio of 1:3. Data were obtained from three independent experiments, and integrated data are shown in the right panel. Values represent the mean ± SD. *P < 0.01. **b** Effects of NTP-GM-D17 on NK cells. NK cells were isolated from expanded PBMCs, and 97% of the prepared cells were CD56^+^/CD3^−^ cells. After treatment with 20 nM NTP-GM-D17 for 5 days, dot blot and graph showed that the number of PD-1^+^ cells decreased to approximately half of the control. Values represent the mean ± SD. *P < 0.01. Data were obtained from three independent experiments. S, NTP-GM-SCR; D, NTP-GM-D17. **c** Cytotoxicity of NK cells to SKOV-3/Luc cells. The experiments were performed with target cells and effector at a ratio of 1:3. Values represent the mean ± SD. *P < 0.01. Data were obtained from three independent experiments. **d** FACS analysis of PD-L1^+^ SKOV-3/Luc-KO cells. Dotted and solid lines indicate data obtained from isotype control IgG and anti-PD-L1 IgG, respectively. **e** Cytotoxicity of NK cells to SKOV-3/Luc-KO cells. The experiments were performed with effector and target cells at a ratio of 3:1. Data were obtained from three independent experiments. Values represent the mean ± SD. NS, not significant. **f** FACS analysis of IFN-γ^+^ NK cells after co-culture with SKOV-3/Luc cells. Dotted and solid lines indicate data obtained from isotype control IgG and anti-IFN-γ IgG, respectively. The right panel showed summarised FACS data from three independent experiments. Values represent the mean ± SD (*n* = 3). *P < 0.01; NS, not significant.

We next investigated whether PBMCs that had been treated with NTP-GM-D17 exerted enhanced cytotoxic activity. As shown in Fig. 4a, PBMCs, which were treated with 20 nM NTP-GM-D17 for 5 days, enhanced apoptosis of RPMI8226 cells, a human myeloma cell line, by twofold (Fig. 4a, *P < 0.01). We then confirmed that NTP-GM-D17 effectively downregulated *PD-1* expression in NK cells that were isolated from expanded PBMCs (Fig. 4b), and observed enhanced cytotoxic activities of these treated cells to SKOV-3/Luc cells (Fig. 4c, *P < 0.01), which expressed a definite level of PD-L1 protein (Fig. 4d, left panel). To prove that an enhancement of tumour-killing activity by NTP-GM-D17 was a result of reduced interaction of PD-1 and PD-L1, the effect of NTP-GM-D17 was examined on a subclone of SKOV-3/Luc cells, the *PD-L1* gene of which was disrupted by the CRISPR/Cas9 system (KO-SKOV-3 cells) (Fig. 4d, right panel). As shown in Fig. 4e, KO-SKOV-3 cells were by themselves susceptible to non-treated NK cells (~50% cells were killed, compare data shown in Fig. 4c), and no enhancement of tumour- killing activity was observed by NTP-GM-D17. Consistent with the observation that NTP-GM-D17 enhanced tumour-killing activity, the numbers of NK cells positive for intracellular IFN-γ increased from 0.8 to 16.1% after treatment with NTP-GM-D17 (Fig. 4f, *P < 0.01: the right panel depicts the integrated data from three experiments).

### NTP-GM-D17-treated NK cells attenuated tumour growth in vivo

**Fig. 5:**
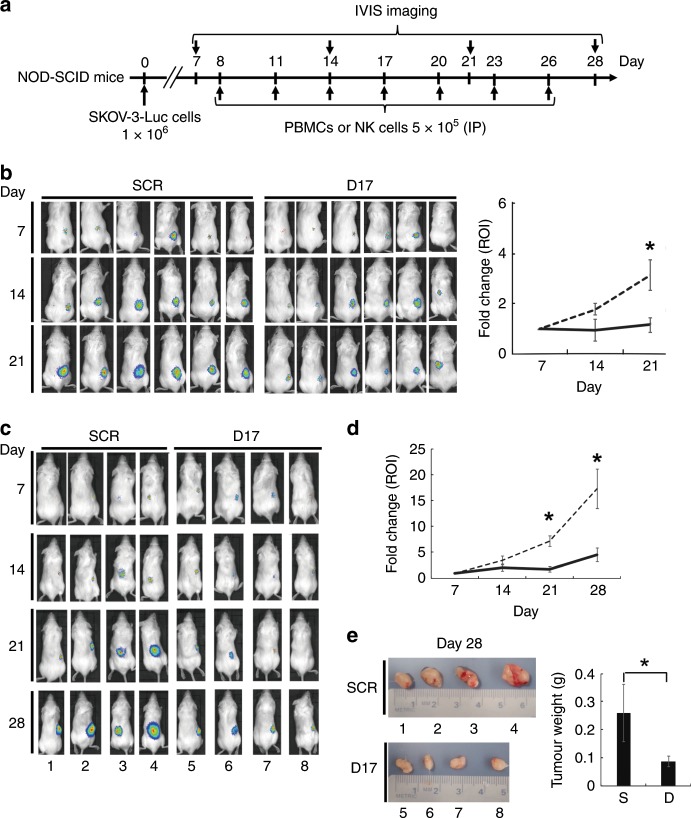
Suppression of tumour growth by NTP-GM-D17-treated cells in vivo. **a** An experimental protocol for assessing antitumour activity in vivo. A schedule of cell injections and IVIS imaging is depicted. IP, intraperitoneal. **b** Suppression of tumour growth by injections of treated PBMCs. Chronological changes of bioluminescence signals from xenografts were shown (right graph). Data from six mice were calculated and compared. Values represent the mean ± SD. ROI, region of interest. *P < 0.01. **c** Suppression of tumour growth by NK cells. **d** Integrated chronological changes of bioluminescence signals from xenografts. Values represent the mean ± SD calculated using data obtained from seven independent mice (data shown in Supplementary Fig. S3C were included into analysis). ROI, region of interest. *P < 0.01. **e** Images of tumour tissues resected on day 28. Graph showing summarised tumour weights of four independent tissues. Values represent the mean ± SD (*n* = 4). S, NTP-GM-SCR; D, NTP-GM-D17. *P < 0.01.

We finally evaluated the antitumour effects of NTP-GM-D17-treated cells. According to an experimental protocol shown in Fig. 5a, we injected SKOV-3/Luc cells to NOD-SCID mice and monitored tumour growth using the IVIS imaging. On day 7 after the injection, we confirmed tumour formation in each mouse (Supplementary Fig. S3A), and randomly divided tumour-bearing mice into two groups, to which two kinds of cells, NTP-GM-D17-treated cells (treated cells) or cells treated with NTP-SCR (control cells), were injected intraperitoneally. First, we confirmed that treated PBMCs significantly inhibited tumour growth on day 21 (Fig. 5b). Then, we focused on purified NK cells and observed that repetitive injections of the treated NK cells effectively attenuated tumour growth. Combined data of two independent experiments using totally seven mice for each group (Supplementary Fig. S3B, C) indicated that injections of the treated NK cells significantly reduced tumour growth, especially from days 21 to 28 (Fig. 5d, *P < 0.01). Direct weight measurement analysis of tumours, which were derived from the four independent mice shown in Fig. 5c, further verified the antitumour effect of the treated NK cells (Fig. 5e, *P < 0.01).

**Fig. 6:**
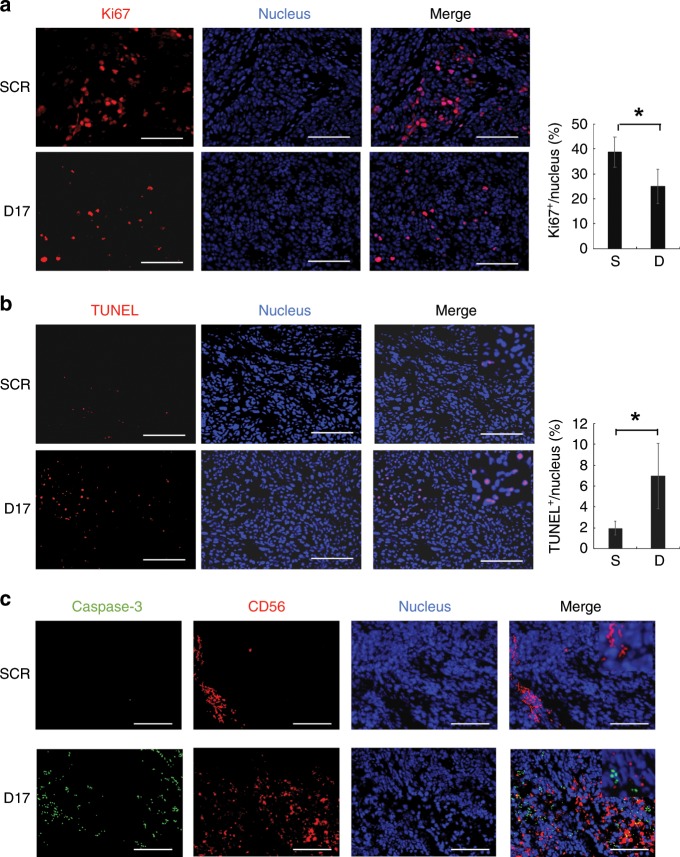
Effects of NTP-GM-D17-treated NK cells on tumour tissues. **a** Immunohistochemical analysis of Ki67^+^ cells in tumour tissues. Nuclei were stained with Hoechst dye. Scale bar, 100 μm (left). The percentage of Ki67^+^ cells was calculated using a Bzx Analyser (KEYENCE) by scanning ~1000 cells in 30–40 images. The same analyses were performed on three independent tumour tissues, and obtained data were integrated. Values represent the mean ± SD (right). S, NTP-GM-SCR; D, NTP-GM-D17. *P < 0.01. **b** TUNEL assay in tumour tissues. The percentage of TUNEL^+^ cells was calculated according to the same procedures depicted in **e**. Values represent the mean ± SD (right). **c** Immunohistochemical analysis of cleaved caspase-3 (green) and CD56 (red).

Immunohistochemical analysis of the sectioned xenografts, which were resected from NOD-SCID mice that were administered NTP-GM-D17-treated NK cells, indicated that the number of Ki67^+^ cells was reduced (Fig. 6a, *P < 0.01), whereas TUNEL^+^ cells were increased (Fig. 6b, *P < 0.01). In addition, the number of CD56^+^ cells and cells positive for cleaved caspase-3 was increased (Fig. 6c). Fine examination suggested that TUNEL^+^ cells were not merged with CD56^+^ cells, suggesting that NTP-GM-D17 enhanced the antitumour activity of NK cells and suppressed tumour growth by inducing apoptosis.

## Discussion

In this study, we established a protein-based GM system by which the endogenous expression of a target gene can be transiently repressed by an exogenously added recombinant protein. The system is composed of a chimeric protein of TALE and DNMT3A/3L tagged with NTP, a nuclear-trafficking peptide. As an initial trial, we currently chose the *PD-1* gene as a target,^27,28^ and treatment of NK cells with NTP-GM-D17 efficiently repressed the mRNA and protein expression of PD-1, with a concomitant enhancement of their antitumour effects both in vitro and in vivo. The downregulation of gene expression by NTP-GM-D17 was attributable to DNA methylation of CpG islands in the promoter region of *PD-1* by DNMT3A/3L, because a mutant NTP-GM with no DNMT3A/3L catalytic activity was unable to induce significant repression of *PD-1* expression (Fig. 2c, Supplementary Fig. S2D).

NTP is a cell-penetrating peptide with potent activity that is composed of ten amino acids derived from viral protein R of human immunodeficiency virus type 1.^19^ Notably, NTP has nuclear-trafficking activity and is especially suitable for protein-based transcriptional regulators. In our previous work, we successfully generated mouse-induced pluripotent stem cell-like clones using NTP–ATF protein. Although NTP–ATF was ~80 kDa in size, we here proved that NTP-GM-D17 of ~150 kDa in size (80 kDa for TALE and 70 kDa for DNMT3A/3L) was also functional, implying that NTP is a powerful tool for trafficking large molecules and manipulating endogenous gene expression.

It has been reported that anti-PD-1 therapy often induces immune-related adverse events^29^ with fatal side effects such as type 1 diabetes mellitus.^8,9^ Moreover, there is an increasing number of reports indicating that the general administration of an anti-PD-1 antibody can induce tumour hyper-progression by augmenting regulatory T cells.^30,31^ Here we showed that the effects of NTP-GM-D17 were transient, and *PD-1* expression in human PBMCs was restored to control levels on day 7 after the final treatment with the protein. Consistent with our previous work showing that the turnover of incorporated NTP–ATF protein was quite urgent,^19^ western blot analysis of PBMCs revealed that NTP-GM protein, once incorporated into the cells, is degraded quickly, and the effects of carried-over proteins were negligible (Fig. 3h). In the current study, we injected 5 × 10^5^ cells seven times (total 3.5 × 10^6^ cells) into in vivo experiments. In preceding studies, the antitumour effects were reported by injecting 10^6^ NK cells twice or single injection of 10^7^ cells to a mouse.^32,33^ No toxic effects were mentioned in both reports, suggesting that NK cells of 2 × 10^6^–10^7^ cells per mouse can exert antitumour effects without toxic phenotypes. In addition, we observed no apparent tumour formation in 15 weeks after the last injection of the treated NK cells (Supplementary Fig. S4A). These observations suggest that NK cells treated with NTP-GM system have less side effects, and that cancer patients could possibly tolerate repeated administrations of NK cells or engineered cancer-targeting T cells that are treated with the protein.

An additional issue with anti-PD-1 therapy is that its positive effects are observed in only ~20% of patients,^1^ suggesting that other IC-related molecules are co-expressed in tumours and interfere with the antitumour effects of immune cells. Of note, the expression of IC molecules, including TIM3 and CEACAM1, has been reported to be enhanced in T and NK cells during cellular expansion.^34^ These observations suggest that combined immunotherapies using multiple antibodies to IC molecules would be more effective, but the simultaneous administration of multiple antibodies would increase the possibility of severe side effects. As TALEs are designable and the NTP-GM system can be applied to different molecules, it is plausible that adoptive immunotherapy combined with multiple NTP-GMs would give a favourable outcome for tumour suppression. In the current study, NTP-GM-D17 efficiently attenuated tumour growth in vivo, but could not completely eliminate tumour cells. It is worthwhile trying to identify additional IC molecules in residual tumours and examining whether the combined usage of NTP-GM-D17 and a novel NTP-GM that targets a different IC molecule can completely eliminate tumour cells. As deep RNA-sequencing data of tumours are now available, it is possible to identify target IC molecules in each patient. If a panel of NTP-GMs, that is, a library of NTP-GMs that can downregulate any target IC molecule, is available in the future, it would be possible to provide personalised adoptive immunotherapy by combining appropriate NTP-GMs based on the obtained expression profiles. Such an approach would hopefully provide a chance for the complete elimination of tumour cells in patients.

## Supplementary information

Supplementary flie

## Data Availability

All data presented within the article and its supplementary information are available upon request from the corresponding author.
