# Supplementary material for: Development of a protein-based system for transient epigenetic repression of immune checkpoint molecule and enhancement of antitumour activity of natural killer cells
Source: Br J Cancer. 2020 Jan 21;122(6):823–34. doi: 10.1038/s41416-019-0708-y (PMC7078285; doi:10.1038/s41416-019-0708-y)

Supplementary Table 1.
Sequences of each cDNA of TALE DNA-binding repeats.

SCR	CTGACCCCAGACCAAGTTGTCGCGATTGCAAGCAACAACGGAGGCAAACAAG<br>CCTTAGAAACAGTCCAGAGATTGTTGCCGGTGCTGTGCCAAGACCACGGC<br>CTGACCCCAGAACAAGTTGTCGCGATTGCAAGCAACAACGGAGGCAAACAAG<br>CCTTAGAAACAGTCCAGAGATTGTTGCCGGTGCTGTGCCAAGCCCACGGC<br>CTGACCCCGGACCAGGTGGTTGCAATCGCGTCACACGATGGGGGAAAGCAGG<br>CCCTAGAAACCGTTCAGCGACTCCTGCCCGTCCTGTGCCAGGCCCACGGC<br>CTGACCCCCGCCCAGGTTGTCGCTATTGCTAGTAACGGCGGAGGCAAACAGG<br>CGCTGGAAACAGTTCAGCGCCTCTTGCCGGTCTTGTGTCAGGACCACGGC<br>CTGACCCCGGACCAGGTGGTTGCAATCGCGTCACACGATGGGGGAAAGCAGG<br>CCCTAGAAACCGTTCAGCGACTCCTGCCCGTCCTGTGCCAGGACCACGGC<br>CTGACCCCGGAACAGGTGGTTGCAATCGCGTCACACGATGGGGGAAAGCAGG<br>CCCTAGAAACCGTTCAGCGACTCCTGCCCGTCCTGTGCCAGGCCCACGGC<br>CTGACCCCGGACCAGGTGGTTGCAATCGCGTCACACGATGGGGGAAAGCAGG<br>CCCTAGAAACCGTTCAGCGACTCCTGCCCGTCCTGTGCCAGGCCCACGGC<br>CTGACCCCAGCCCAGGTTGTGGCCATCGCCAGCAACATAGGTGGCAAGCAGG<br>CCCTCGAAACCGTCCAGAGACTGTTACCGGTTCTCTGCCAGGACCACGGC<br>CTGACCCCGGACCAGGTGGTTGCAATCGCGTCACACGATGGGGGAAAGCAGG<br>CCCTAGAAACCGTTCAGCGACTCCTGCCCGTCCTGTGCCAGGACCACGGC<br>CTGACCCCAGAACAGGTTGTGGCCATCGCCAGCAACATAGGTGGCAAGCAGG<br>CCCTCGAAACCGTCCAGAGACTGTTACCGGTTCTCTGCCAGGCCCACGGC<br>CTGACCCCGGACCAGGTGGTTGCAATCGCGTCACACGATGGGGGAAAGCAGG<br>CCCTAGAAACCGTTCAGCGACTCCTGCCCGTCCTGTGCCAGGCCCACGGC<br>CTGACCCCGGCCCAGGTGGTTGCAATCGCGTCACACGATGGGGGAAAGCAGG<br>CCCTAGAAACCGTTCAGCGACTCCTGCCCGTCCTGTGCCAGGACCACGGC<br>CTGACCCCGGACCAGGTGGTTGCAATCGCGTCACACGATGGGGGAAAGCAGG<br>CCCTAGAAACCGTTCAGCGACTCCTGCCCGTCCTGTGCCAGGACCACGGC<br>CTGACCCCCGAACAGGTTGTCGCTATTGCTAGTAACGGCGGAGGCAAACAGG<br>CGCTGGAAACAGTTCAGCGCCTCTTGCCGGTCTTGTGTCAGGCCCACGGC<br>CTGACCCCGGACCAGGTGGTTGCAATCGCGTCACACGATGGGGGAAAGCAGG<br>CCCTAGAAACCGTTCAGCGACTCCTGCCCGTCCTGTGCCAGGCCCACGGC<br>CTGACCCCAGCCCAAGTTGTCGCGATTGCAAGCAACAACGGAGGCAAACAAG<br>CCTTAGAAACAGTCCAGAGATTGTTGCCGGTGCTGTGCCAAGACCACGGC
D17	CTGACCCCGGACCAGGTGGTTGCAATCGCGTCACACGATGGGGGAAAGCAGG<br>CCCTAGAAACCGTTCAGCGACTCCTGCCCGTCCTGTGCCAGGACCACGGC<br>CTGACCCCGGAACAGGTGGTTGCAATCGCGTCACACGATGGGGGAAAGCAGG<br>CCCTAGAAACCGTTCAGCGACTCCTGCCCGTCCTGTGCCAGGCCCACGGC<br>CTGACCCCGGACCAGGTGGTTGCAATCGCGTCACACGATGGGGGAAAGCAGG<br>CCCTAGAAACCGTTCAGCGACTCCTGCCCGTCCTGTGCCAGGCCCACGGC<br>CTGACCCCCGCCCAGGTTGTCGCTATTGCTAGTAACGGCGGAGGCAAACAGG<br>CGCTGGAAACAGTTCAGCGCCTCTTGCCGGTCTTGTGTCAGGACCACGGC<br>CTGACCCCCGACCAGGTTGTCGCTATTGCTAGTAACGGCGGAGGCAAACAGG<br>CGCTGGAAACAGTTCAGCGCCTCTTGCCGGTCTTGTGTCAGGACCACGGC<br>CTGACCCCGGAACAGGTGGTTGCAATCGCGTCACACGATGGGGGAAAGCAGG<br>CCCTAGAAACCGTTCAGCGACTCCTGCCCGTCCTGTGCCAGGCCCACGGC<br>CTGACCCCAGACCAGGTTGTGGCCATCGCCAGCAACATAGGTGGCAAGCAGG<br>CCCTCGAAACCGTCCAGAGACTGTTACCGGTTCTCTGCCAGGCCCACGGC<br>CTGACCCCAGCCCAGGTTGTGGCCATCGCCAGCAACATAGGTGGCAAGCAGG<br>CCCTCGAAACCGTCCAGAGACTGTTACCGGTTCTCTGCCAGGACCACGGC<br>CTGACCCCGGACCAGGTGGTTGCAATCGCGTCACACGATGGGGGAAAGCAGG<br>CCCTAGAAACCGTTCAGCGACTCCTGCCCGTCCTGTGCCAGGACCACGGC<br>CTGACCCCGGAACAGGTGGTTGCAATCGCGTCACACGATGGGGGAAAGCAGG<br>CCCTAGAAACCGTTCAGCGACTCCTGCCCGTCCTGTGCCAGGCCCACGGC<br>CTGACCCCCGACCAGGTTGTCGCTATTGCTAGTAACGGCGGAGGCAAACAGG<br>CGCTGGAAACAGTTCAGCGCCTCTTGCCGGTCTTGTGTCAGGCCCACGGC<br>CTGACCCCAGCCCAAGTTGTCGCGATTGCAAGCAACAACGGAGGCAAACAAG<br>CCTTAGAAACAGTCCAGAGATTGTTGCCGGTGCTGTGCCAAGACCACGGC<br>CTGACCCCAGACCAGGTTGTGGCCATCGCCAGCAACATAGGTGGCAAGCAGG<br>CCCTCGAAACCGTCCAGAGACTGTTACCGGTTCTCTGCCAGGACCACGGC<br>CTGACCCCGGAACAGGTGGTTGCAATCGCGTCACACGATGGGGGAAAGCAGG<br>CCCTAGAAACCGTTCAGCGACTCCTGCCCGTCCTGTGCCAGGCCCACGGC<br>CTGACCCCGGACCAGGTGGTTGCAATCGCGTCACACGATGGGGGAAAGCAGG<br>CCCTAGAAACCGTTCAGCGACTCCTGCCCGTCCTGTGCCAGGCCCACGGC<br>CTGACCCCCGCCCAGGTTGTCGCTATTGCTAGTAACGGCGGAGGCAAACAGG<br>CGCTGGAAACAGTTCAGCGCCTCTTGCCGGTCTTGTGTCAGGACCACGGC

D17 cDNA encoding TALE molecule designed to target the *PD-1* promoter sequence. Scrambled (SCR) TALE which recognized TGGCTCCCACACCCTCG used as a control.

Supplementary Table 2.
Sequences of primers for RT-PCR.

GAPDH	Forward	ACAACTTTGGTATCGTGGAAGG
	Reverse	GCCATCACGCCACAGTTTC
PD-1	Forward	AAGTTTCAGGGAAGGTCAG
	Reverse	CTGGGCATGTGTAAAGGT

Supplementary Table 3.
Sequences of sgRNA.

PD-L1 sgRNA 1	GATCCCCGACCGCTGCATGATCAGCTAGTTTTAGAGCTAGAAATAGCAAGTTAAA<br>AAAGGCTAGTCCGTTATCAACTTGAAAAAGTGGCACCGAGTCGGTGCTTTTTTA
PD-L1 sgRNA 2	GATCCCCGGCTGCATGATCAGCTATGGGTTTTAGAGCTAGAAATAGCAAGTTAAA<br>AAAGGCTAGTCCGTTATCAACTTGAAAAAGTGGCACCGAGTCGGTGCTTTTTTA

SUPPLEMENTARY FIGURES

Supplementary Figure S1

(A) Schematic structure of pEU-NTP-GM for expression in a cell-free protein expression system. Each NTP-GM protein was expressed with wheat germ extract and purified by glutathione beads (open arrow in left panel). After treatment with PreScission protease, the cleaved end-product (solid arrow in right panel) was confirmed and used for a DNA methylation assay. CBB protein staining images are shown. (B) *PD-1* mRNA expression in CEM, Jurkat, and MOLT-4 cells. Expression levels were normalized to *GAPDH* mRNA expression. Values represent the mean ± SD. Three independent experiments were performed. (C) Bisulfite sequencing analysis of CR-C and CR-B from non-treated Jurkat, MOLT-4, and CEM cells.

Supplementary Figure S2

(A) Effects of NTP-GM-D17 on *PD-1* mRNA expression on primary PBMCs. Expression levels were normalized to *GAPDH* mRNA expression. Values represent the mean ± SD of data obtained from three independent experiments. (B) NTP-GM-D17 represses PD-1

expression in primary PBMCs. Representative results of FACS analysis of the expression of PD-1 and CD3 are shown. (C) Expansion of PBMCs increases the number of PD-1$^+$ cells. PBMCs were cultured for 14 days in the presence of anti-CD3, anti-CD16 and IL-2, and subjected to FACS analysis. (D) Effects of NTP-GM-D17^{E752A} on *PD-1* mRNA expression. Expression levels were normalized to *GAPDH* mRNA expression. Values represent the mean ± SD of three independent experiments. (E) No toxic effects of 1, 10, and 20 nM NTP-GM protein on PBMCs. Cytotoxicity assay was done by measuring LDH in the culture supernatant of PBMCs treated for 5 days with NTP-GM or NTP-SCR proteins. (F) NTP-GM-D17 reduces *PD-1* mRNA expression in PBMCs. PBMCs were treated for 5 days with NTP-GM-D17, and expression levels were evaluated at day 6, 9 and 12 and normalized to the level of *GAPDH* mRNA. Data are shown as the mean ± SD (n = 3). N, non-treated; S, NTP-GM-SCR; D, NTP-GM-D17.

Supplementary Figure S3

(A) Tumor size monitored on day 7 after tumor inoculation. Luminescence signals of total 7 mice, which were used in experiments shown in Figure 5C and Supplementary Figure

S3B, were integrated for analysis**.** No significant difference was observed in two groups of mice injected with control NK cells or treated NK cells. (B) IVIS imaging of mice injected the treated NK cells. (C) Integrated data of luminescence signals. Data of chronological monitoring by the IVIS imaging system on these three mice were analyzed. Tumor-bearing mice were injected with the treated NK cells. ROI, region of interest.

Supplementary Figure S4

(A) No apparent tumor formation by NTP-GM-D17. Ventrotomy images of mice injected with treated NK cells (protocol shown in Figure 5A) after 15 weeks from last injection are shown. NT, non-treated mouse. (B) No remarkable increase of white blood cell count in mice injected with treated NK cells. Numbers of white blood cells were counted.

Supplementary Figure S1

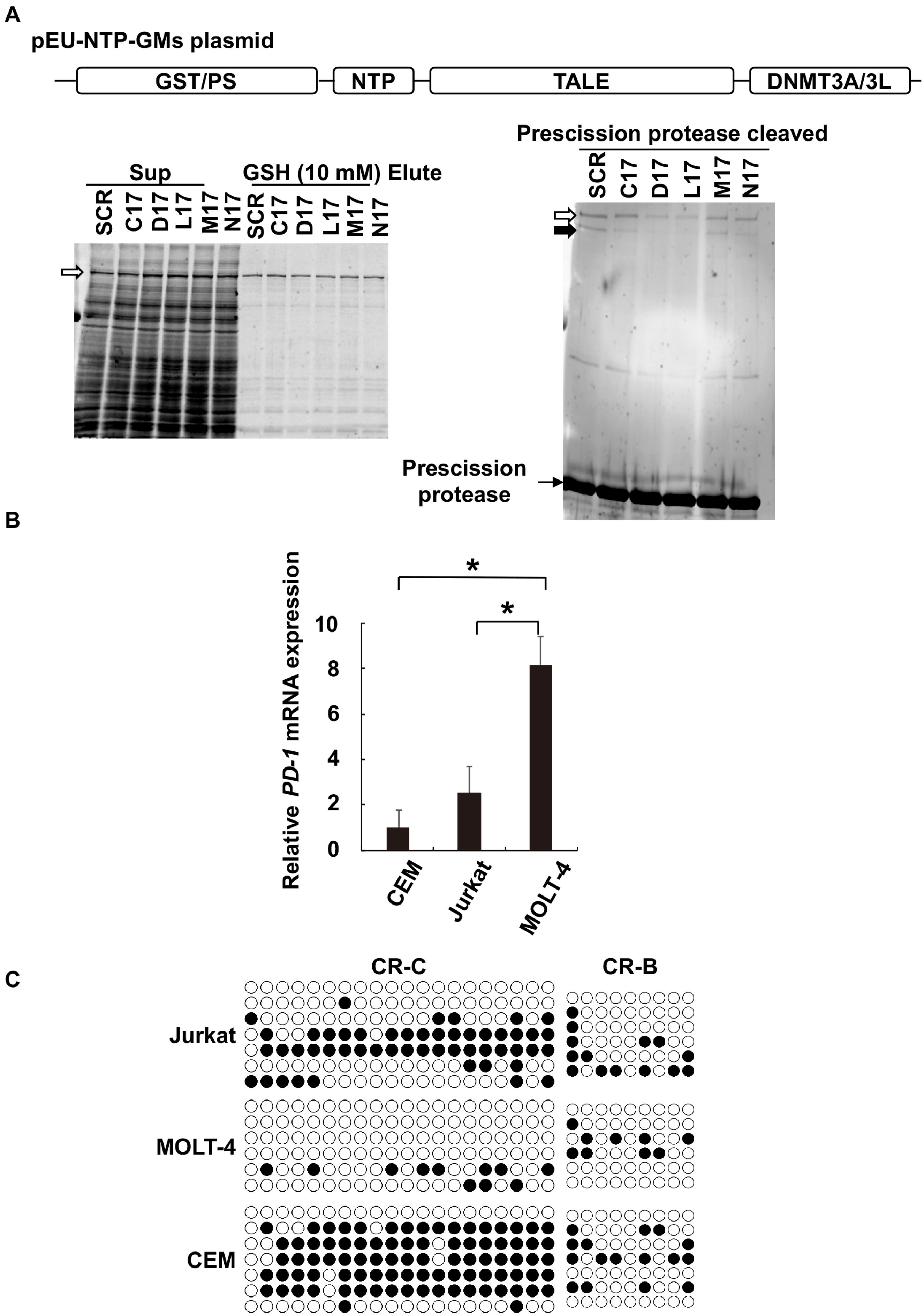

Supplementary Figure S2

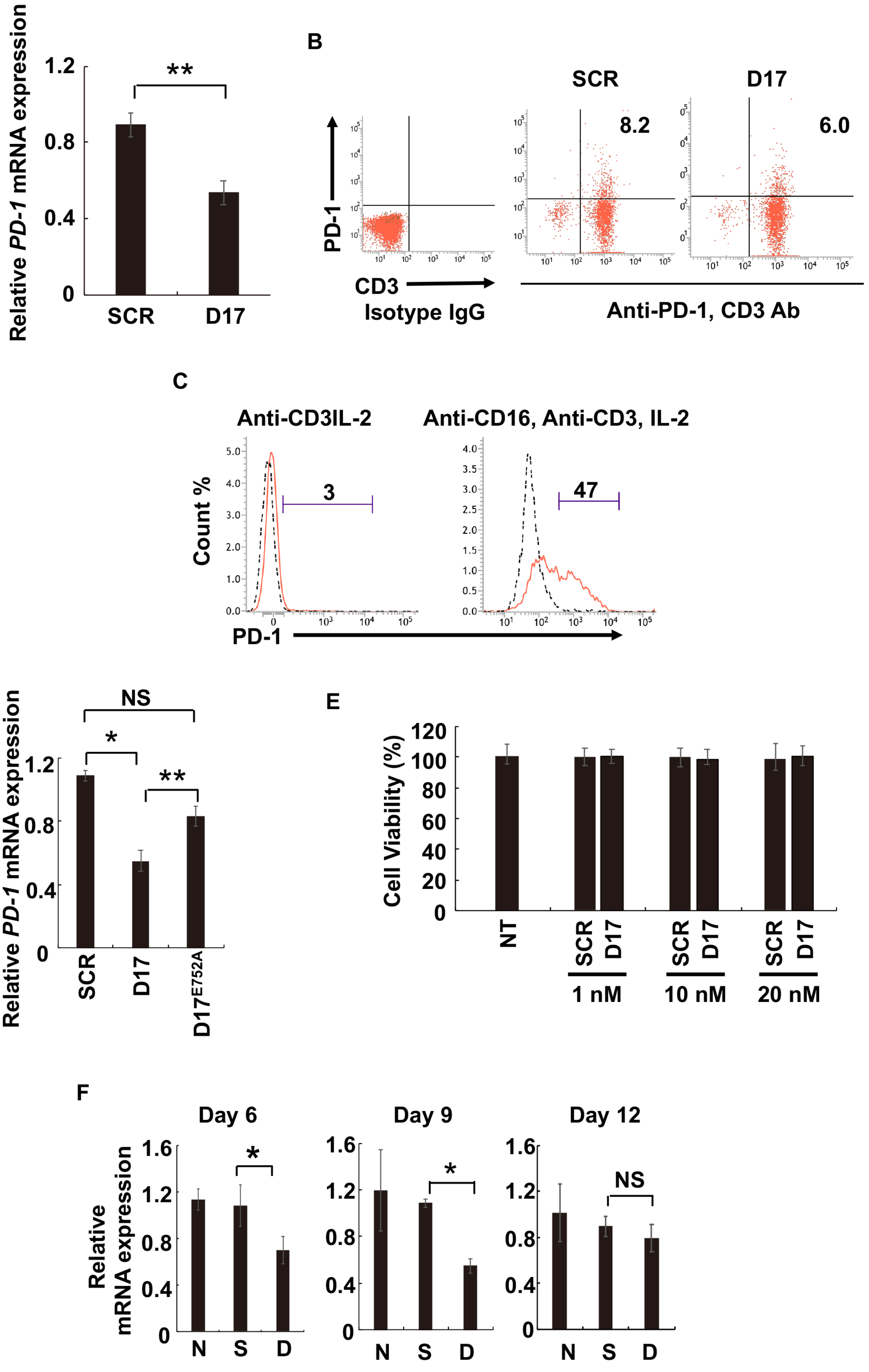

Supplementary Figure S3

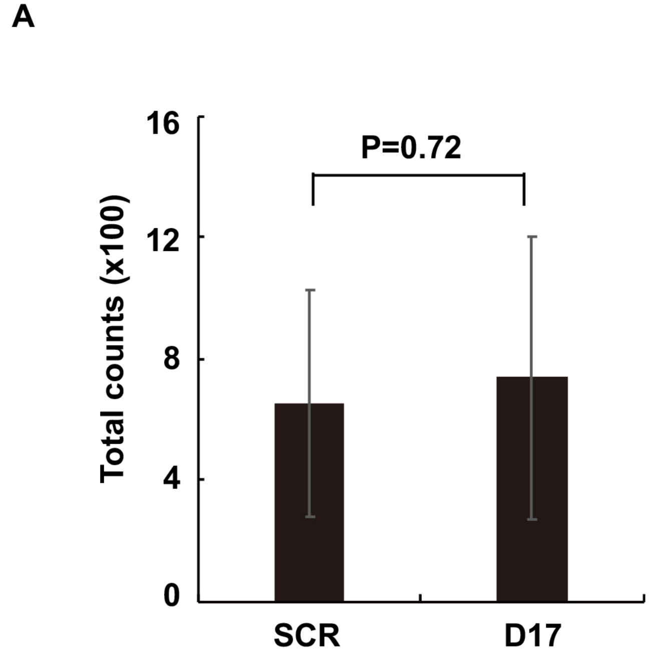

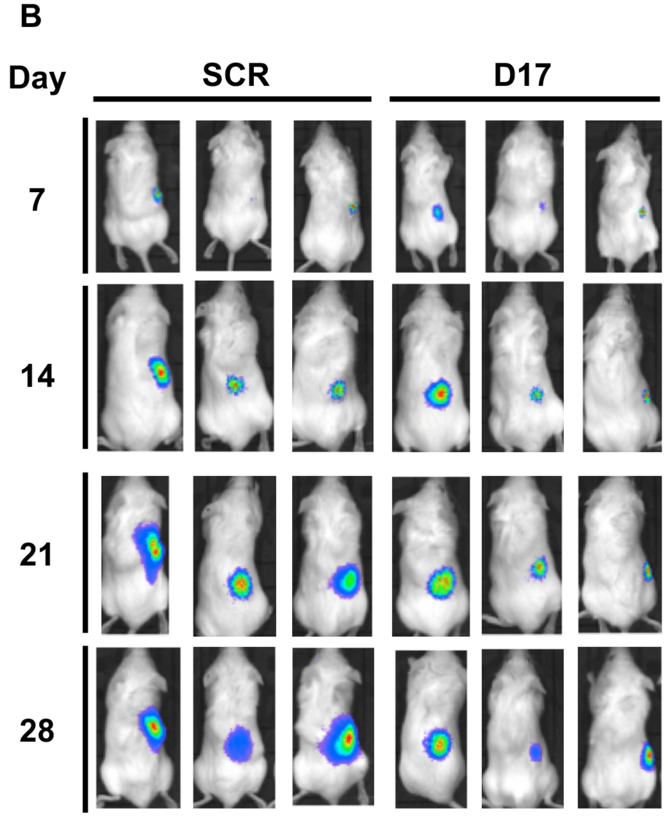

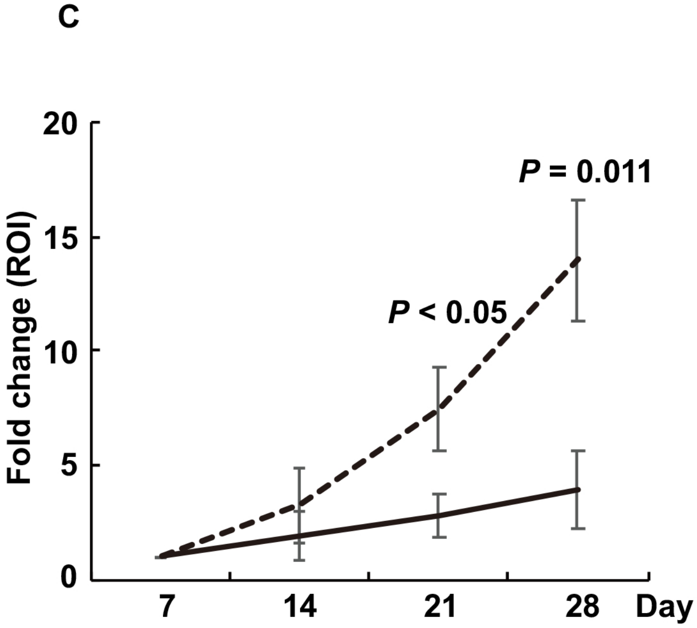

Supplementary Figure S4

A

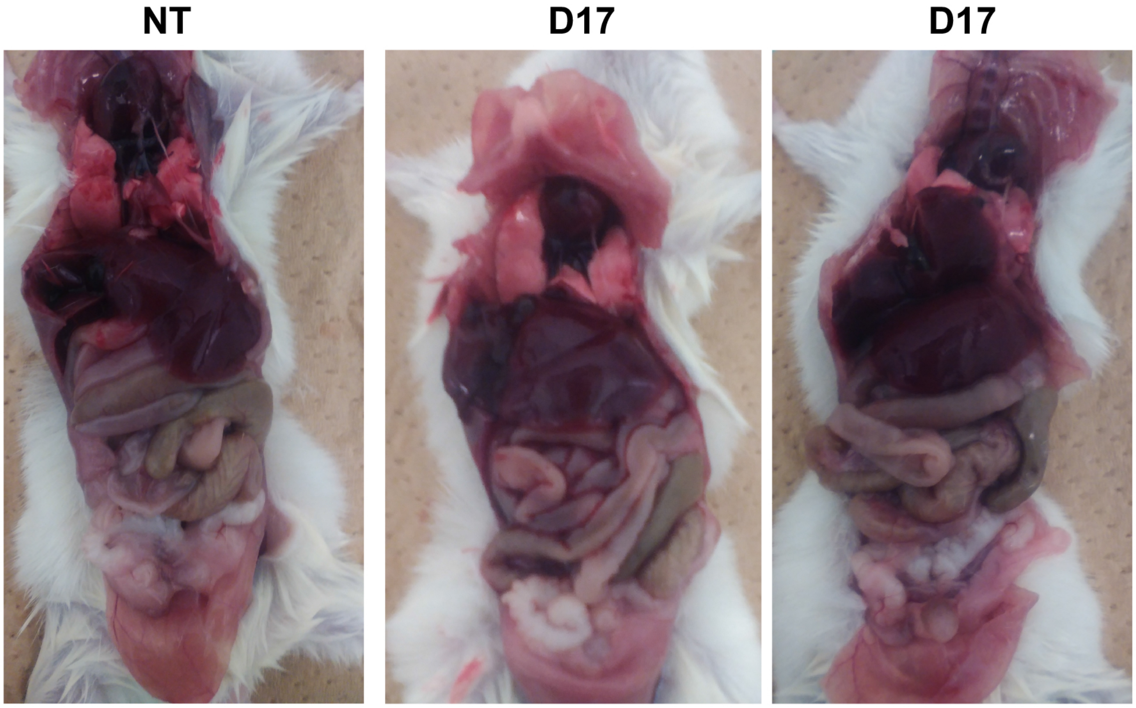

B

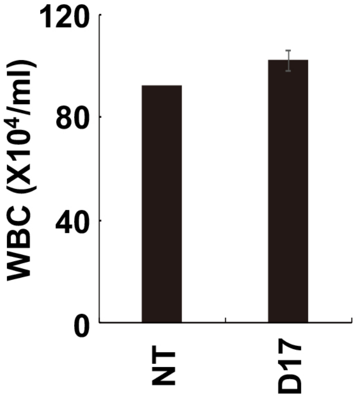

Supplement: Supplementary file 1 — Supplementary flie [file 41416_2019_708_MOESM1_ESM.pdf]
